# An In Vivo Dual-Observation Method to Monitor Tumor Mass and Tumor-Surface Blood Vessels for Developing Anti-Angiogenesis Agents against Submillimeter Tumors

**DOI:** 10.3390/ijms242417234

**Published:** 2023-12-07

**Authors:** Tomoko Tachibana, Tomoko Gowa Oyama, Yukie Yoshii, Fukiko Hihara, Chika Igarashi, Mitsuhiro Shinada, Hiroki Matsumoto, Tatsuya Higashi, Toshihiko Kishimoto, Mitsumasa Taguchi

**Affiliations:** 1 Department of Molecular Imaging and Theranostics, National Institutes for Quantum Science and Technology (QST), Chiba 263-8555, Japan; fukiko.hihara@gmail.com (F.H.); cigaras2023@gmail.com (C.I.); 7123001s@st.toho-u.ac.jp (M.S.); hirocky01.star64@gmail.com (H.M.); higashi.tatsuya@qst.go.jp (T.H.); 2 Faculty of Science, Toho University, Chiba 274-8510, Japan; kisimoto@biomol.sci.toho-u.ac.jp; 3 Foundational Quantum Technology Research Directorate, National Institutes for Quantum Science and Technology (QST), Gunma 370-1292, Japan; ohyama.tomoko@qst.go.jp (T.G.O.); taguchi.mitsumasa@qst.go.jp (M.T.); 4 Visible Cancer Drug Research Unit, National Institutes for Quantum Science and Technology (QST), Chiba 263-8555, Japan

**Keywords:** in vivo, angiogenesis, radiation-crosslinked gelatin hydrogel microwell array, spheroid, subcutaneous submillimeter tumor xenograft

## Abstract

Managing metastasis at the early stage and detecting and treating submillimeter tumors at early metastasis are crucial for improving cancer prognosis. Angiogenesis is a critical target for developing drugs to detect and inhibit submillimeter tumor growth; however, drug development remains challenging because there are no suitable models for observing the submillimeter tumor mass and the surrounding blood vessels in vivo. We have established a xenograft subcutaneous submillimeter tumor mouse model with HT-29-RFP by transplanting a single spheroid grown on radiation-crosslinked gelatin hydrogel microwells. Here, we developed an in vivo dual-observation method to observe the submillimeter tumor mass and tumor-surface blood vessels using this model. RFP was detected to observe the tumor mass, and a fluorescent angiography agent FITC-dextran was administered to observe blood vessels via stereoscopic fluorescence microscopy. The anti-angiogenesis agent regorafenib was used to confirm the usefulness of this method. This method effectively detected the submillimeter tumor mass and tumor-surface blood vessels in vivo. Regorafenib treatment revealed tumor growth inhibition and angiogenesis downregulation with reduced vascular extremities, segments, and meshes. Further, we confirmed that tumor-surface blood vessel areas monitored using in vivo dual-observation correlated with intratumoral blood vessel areas observed via fluorescence microscopy with frozen sections. In conclusion, this method would be useful in developing anti-angiogenesis agents against submillimeter tumors.

## 1. Introduction

Cancer remains the leading cause of death worldwide, and metastasis is estimated to cause 90% of cancer deaths [1,2]. The management of metastasis at the early stage and the effective detection and treatment of submillimeter tumors at early metastasis is necessary to improve cancer prognosis. Angiogenesis is an essential step in submillimeter tumor growth at early metastasis. It is known that tumor cell spheroids cultured in vitro have an upper size limit owing to a lack of nutrient diffusion from the media into the spheroid core [3,4]. Similarly, tumors in vivo have growth limitations because of restricted nutrient diffusion from the nearest blood vessels, which are approximately 100–500 μm, unless angiogenesis occurs [5,6]. As the submillimeter tumors reach their growth limitations, the tumor core becomes hypoxic, leading to the upregulation of angiogenic growth factors, such as vascular endothelial growth factor (VEGF) families and fibroblast growth factor members [7,8]. Consequently, new blood vessels are derived from the nearest blood vessels through the construction of branches that make vascular interconnections and loops, thereby covering the submillimeter tumors [9,10]. Thus, angiogenesis is a critical target to inhibit tumor growth in early metastasis [11,12,13,14]. To evaluate the angiogenesis of submillimeter tumors and develop agents against them, the monitoring of tumor-surface blood vessels and angiogenesis parameters, such as vascular extremities, segments, and meshes, is needed.

Many anti-angiogenesis agents have been preclinically and clinically developed. VEGF and its receptor (VEGFR) have been the most studied factors to develop anti-angiogenesis agents. Regorafenib is a clinically approved anti-angiogenesis agent of an oral multikinase inhibitor targeting VEGFR and many related factors [15,16]. Preclinical studies have shown that regorafenib exhibits antitumor activity in colorectal cancer models [17]. Regorafenib was initially approved for the treatment of metastatic colorectal cancer and was later expanded to other tumors. However, the development of anti-angiogenesis agents against submillimeter tumors remains challenging because there is no suitable in vivo observation method for the submillimeter tumor mass and tumor-surface blood vessels. To date, metastatic mouse models constructed via the intravenous or intraperitoneal administration of cancer cell suspension have been used to study submillimeter tumors of metastatic colorectal cancer [18,19,20]. However, the in vivo observation of the submillimeter tumor mass and the regulation of tumor growth are challenging. 

**Figure 1:**
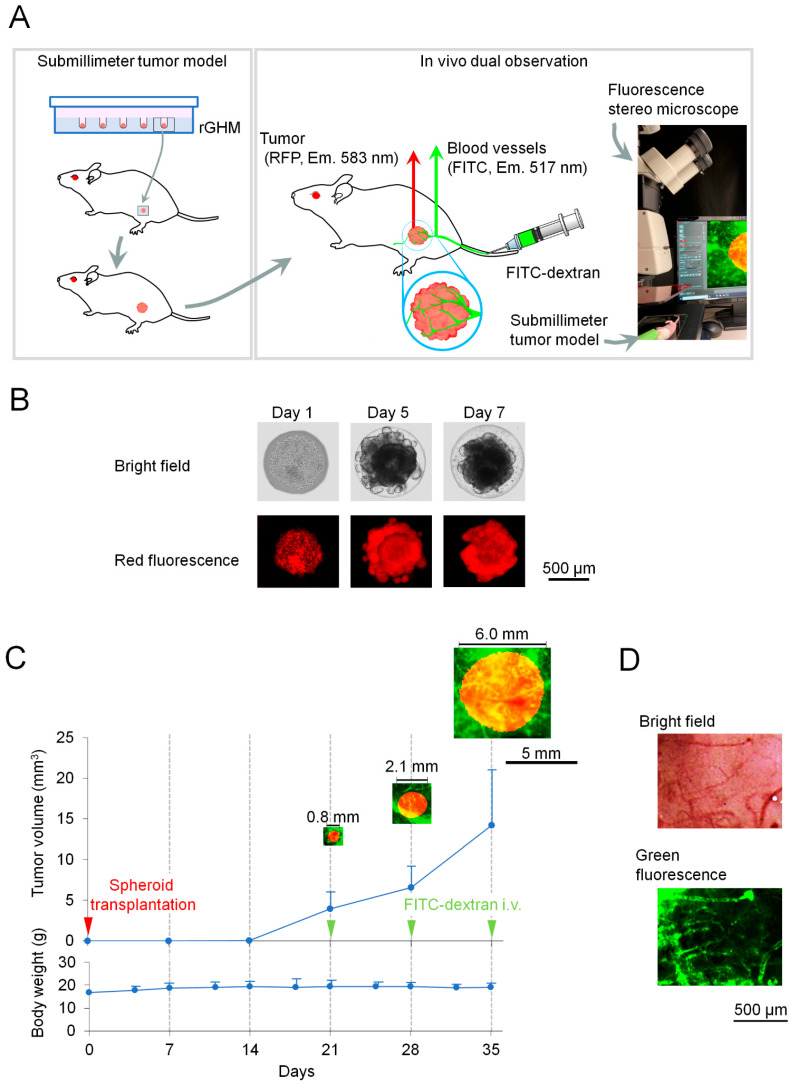
Establishment of in vivo dual-observation method with the submillimeter tumor model. (**A**) Schematic illustration of the submillimeter tumor model and in vivo dual-observation method of tumor mass and tumor-surface blood vessels, using fluorescence stereomicroscopy. To establish the model, HT-29-RFP spheroids were obtained through cultivation on the rGHM array and transplanted into mice with rGHM as a scaffold (left). For in vivo dual-observation, tumor mass was visualized by detecting RFP signals from HT-29-RFP tumors, whereas tumor-surface blood vessels were visualized via intravenous administration of FITC-dextran (right). (**B**) Representative images of spheroid formation by HT-29-RFP cells in rGHM array over time (Days 1, 5, and 7). Images were obtained using an inverted fluorescence microscope, and both bright-field and red fluorescence views are shown in the upper and lower parts of the image, respectively. (**C**) Changes in tumor volume over time. Tumor volume was calculated from the major and minor lengths of tumors obtained through in vivo dual-observation. A single spheroid was transplanted on Day 0 (red arrowhead), and in vivo dual-observation was performed on Days 21, 28, and 35 (green arrowheads) by injecting FITC-dextran. Representative images of tumor and tumor-surface blood vessels at each time point (upper) and body weight changes throughout the experiment (lower) are shown. (**D**) Representative images of tumor-surface blood vessels of tumor samples isolated on Day 35. Images were obtained using a stereoscopic fluorescence microscope. Bright-field and green fluorescence images are shown on the top and bottom, respectively.

To address this gap, we developed a practical xenograft in vivo mouse model of a subcutaneous submillimeter tumor with human colorectal cancer HT-29-RFP (red fluorescent protein) by transplanting a single spheroid grown on radiation-crosslinked gelatin hydrogel microwells (rGHMs) [21]. In this model, subcutaneous submillimeter tumors are easily observable in vivo through the detection of RFP via stereoscopic fluorescence microscopy. This model enables the formation of uniform subcutaneous submillimeter tumor xenografts by using rGHMs as a culture base to construct spheroids and as a biocompatible and biodegradable transplantation scaffold. Using this model, we further developed an in vivo dual-observation method for the practical monitoring of the tumor mass and tumor-surface blood vessels to develop an anti-angiogenesis agent against submillimeter tumors (Figure 1A). We used intravenously administered FITC-dextran as a fluorescent angiography agent to observe tumor-surface blood vessels. We evaluated the usefulness of this method by comparing submillimeter tumors treated with regorafenib to those in the vehicle control. 

## 2. Results

### 2.1. In Vivo Dual-Observation of Submillimeter Tumor Model

HT-29-RFP spheroids were obtained through cultivation on the rGHM array for 7 d (Figure 1B) and transplanted into mice. In this experiment, we observed that, by administering FITC-dextran as a fluorescent angiography agent into the subcutaneous submillimeter tumor model on Day 21 after spheroid transplantation, the tumor mass and tumor-surface blood vessels were effectively visualized (Figure 1C). On Day 21, the tumor area, major length, minor length, and tumor-surface blood vessel area were 0.19 ± 1.96 mm^2^, 0.5 ± 0.32 mm, 0.35 ± 0.27 mm, and 0.06 ± 0.04 mm^2^, respectively (*n* = 5). Thereafter, the tumor mass increased with time (Figure 1C, upper). There was no significant reduction in body weight after FITC-dextran administration, which revealed its low toxicity levels (Figure 1C, lower). We observed the distribution of the tumor-surface blood vessels of tumor samples isolated on Day 35 by obtaining bright-field images using a stereoscopic fluorescence microscope and compared them to those observed in the green fluorescence images using FITC-dextran (Figure 1D). This confirmed that the pattern of blood vessel distribution was consistent using both observation methods.

### 2.2. Evaluation of Comparative Experiment with Regorafenib 

**Figure 2:**
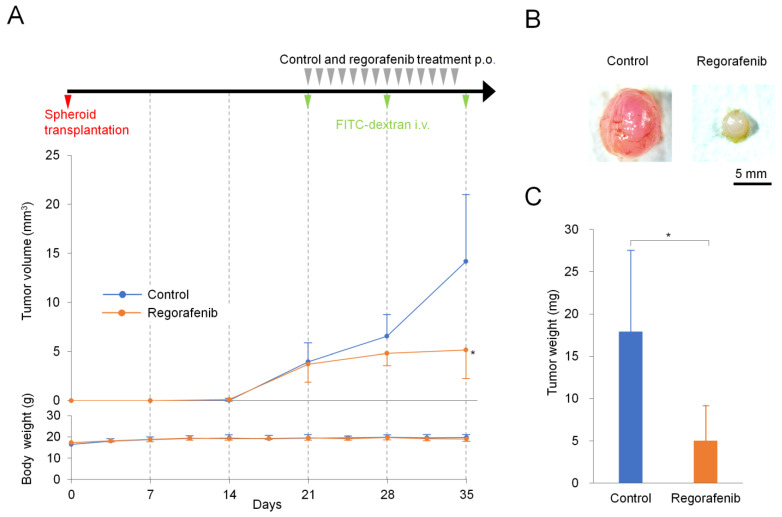
Comparative experiment with regorafenib. (**A**) Schedule of comparative experiment with regorafenib and control groups and the changes in tumor volume and body weight. Timeline of the schedule (upper). A single spheroid was transplanted on Day 0 (red arrowhead). After 21 d, the regorafenib group was orally administered (p.o.) daily with regorafenib, while the control group received vehicle administration in a similar manner (gray arrowheads). To perform in vivo dual-observation, both groups received an intravenous injection of FITC-dextran on Days 21, 28, and 35, using a stereoscopic fluorescence microscope (green arrowheads). Changes in tumor volume over time of control and regorafenib groups (middle). There is a significant difference between the two groups on Day 35 (*, *p* < 0.05). The tumor volume was calculated from the major and minor lengths of tumors obtained through in vivo dual-observation. The body weight changes in control and regorafenib groups throughout the experimental period (lower). There was no significant difference between the two groups. (**B**) Representative images of tumors resected from the submillimeter tumor model on Day 35 for both control and regorafenib groups are shown on the left and right, respectively. (**C**) Weight of excised tumors on Day 35 after implantation in control and regorafenib groups. There is a significant difference between the two groups (*, *p* < 0.05). Data are presented as the mean ± standard deviation (SD).

We further conducted comparative experiments with subcutaneous submillimeter tumor model mice orally administered with regorafenib (10 mg/kg, daily) (regorafenib group) and a vehicle control (control group) (*n* = 10/group). The treatment schedule, tumor volume changes, and body weight changes are depicted in Figure 2A. The regorafenib group showed a significant tumor volume reduction compared to the control on Day 35 (*p* < 0.05). There was no significant reduction in body weight (*p* < 0.05) and no apparent changes in general health status during the experiment in both control and regorafenib groups, which demonstrated that this method did not adversely affect the mice and that regorafenib had negligible toxicity. Figure 2B shows representative images of tumors resected from the subcutaneous submillimeter tumor model on Day 35 for both groups. The tumor weight in isolated tumors on Day 35 had significantly reduced in the regorafenib group compared to the control (Figure 2C) (*p* < 0.05).

**Figure 3:**
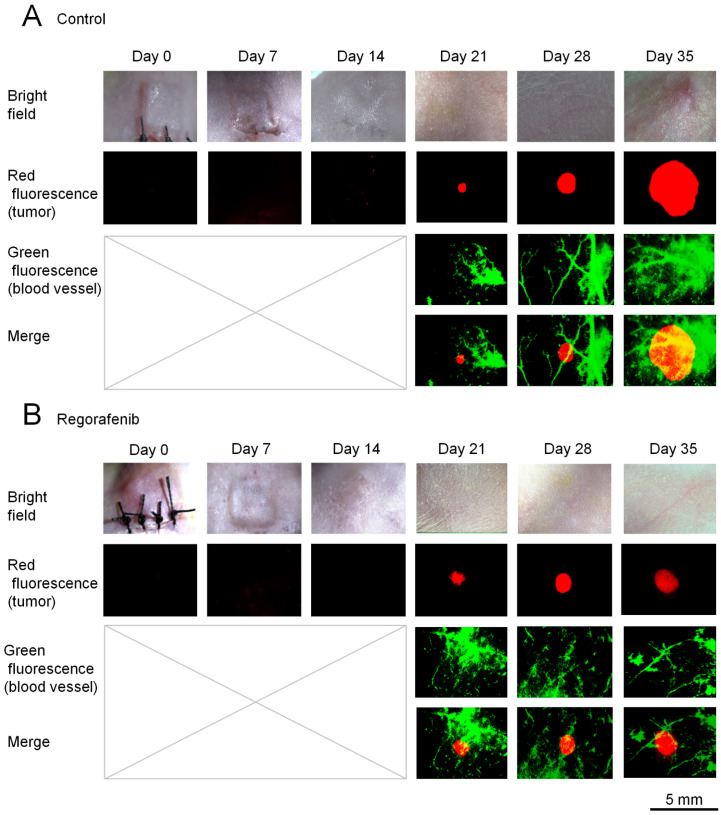
In vivo dual-observation of tumor mass and tumor-surface blood vessels in a comparative experiment with regorafenib. Representative images obtained from in vivo dual-observation of the submillimeter tumor model of the (**A**) control and (**B**) regorafenib groups with time. Bright field (upper row), red fluorescence from RFP of the HT-29-RFP tumors (second row), green fluorescence from tumor-surface blood vessels detected using FITC-dextran (third row), and merge of red fluorescence and green fluorescence (bottom row) views.

**Figure 4:**
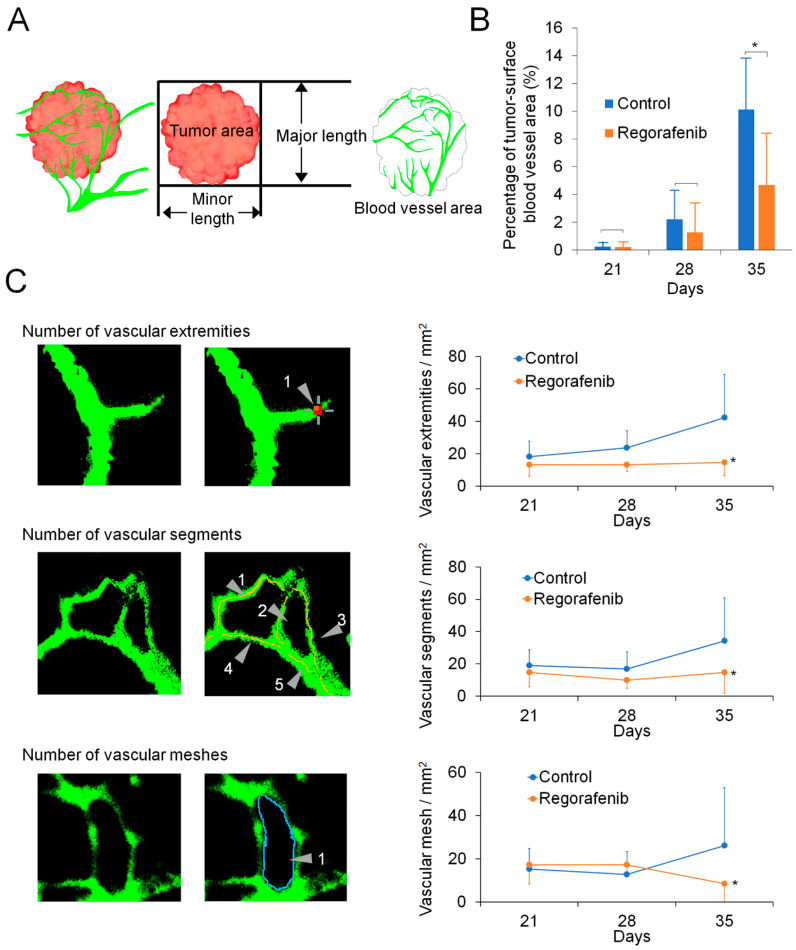
Analysis of tumor-surface blood vessels. (**A**) Schematic illustration of the parameters (tumor area, major length, minor length, and blood vessel area) used in this study. The images of tumor and blood vessel areas were obtained through fluorescence stereoscopic observation, and both areas were calculated using ImageJ software version 1.53. (**B**) Changes in the percentage of blood vessel area on tumor area over time in the control and regorafenib groups detected via in vivo dual-observation. Percentage of blood vessel area (%) = [blood vessel area (mm^2^)/tumor area (mm^2^)] × 100. There were significant differences between the two groups on Day 35 (*, *p* < 0.05). Data represent the mean ± SD. (**C**) Observation of angiogenesis parameters. Representative original images (left column). Images with symbols for the analysis of each parameter (middle column). Changes in the number of each parameter (right column). There were significant differences between the two groups on Days 35 (*, *p* < 0.05). Data represent the mean ± SD.

Figure 3 shows representative images obtained from in vivo dual-observation over time after spheroid transplantation. Figure 4 shows the quantitative analysis of the tumor-surface blood vessel area (%) and angiogenesis parameters, such as vascular extremities, segments, and meshes. In the control group, the tumor volume increased with time, while, in the regorafenib group, the tumor volume stayed constant over time. The percentage of the tumor-surface blood vessel area (%) tended to decrease one week after regorafenib treatment (Day 28) against submillimeter tumors, and there was a significant reduction after 2 weeks (Day 35) (Figure 3A,B and Figure 4A,B) (*p* < 0.05). Figure 4C depicts the analyses of angiogenesis parameters. Similarly, we observed a tendency towards a reduction in vascular extremities and segments on Day 28, and significant reductions in vascular extremities, segments, and meshes on Day 35 (*p* < 0.05). 

### 2.3. Frozen Section Analysis

**Figure 5:**
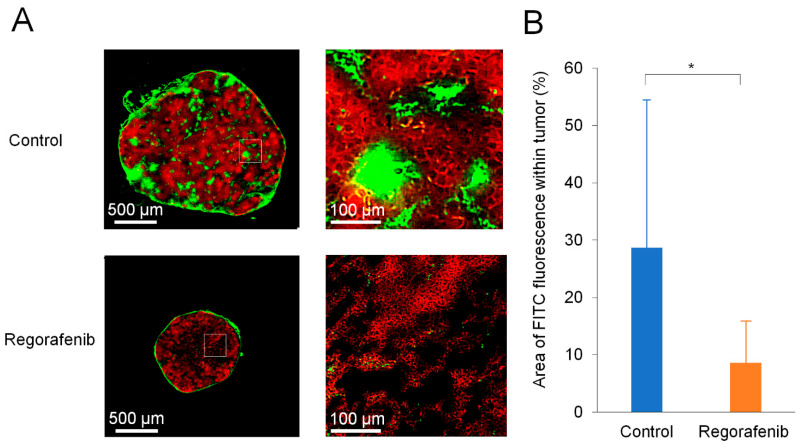
Fluorescence microscopic observation with frozen sections of resected tumors on Day 35 in the comparative study. (**A**) Representative images obtained from the control (upper) and regorafenib (lower) groups. The images show red fluorescence from RFP of tumor area and green fluorescence from blood vessel areas stained with FITC-dextran. The images on the right are enlarged views of the white squares in the images on the left. (**B**) Percentage of blood vessel area on tumor area observed with frozen sections for the control and regorafenib groups. There is a significant difference between the two groups (*, *p* < 0.05).

**Figure 6:**
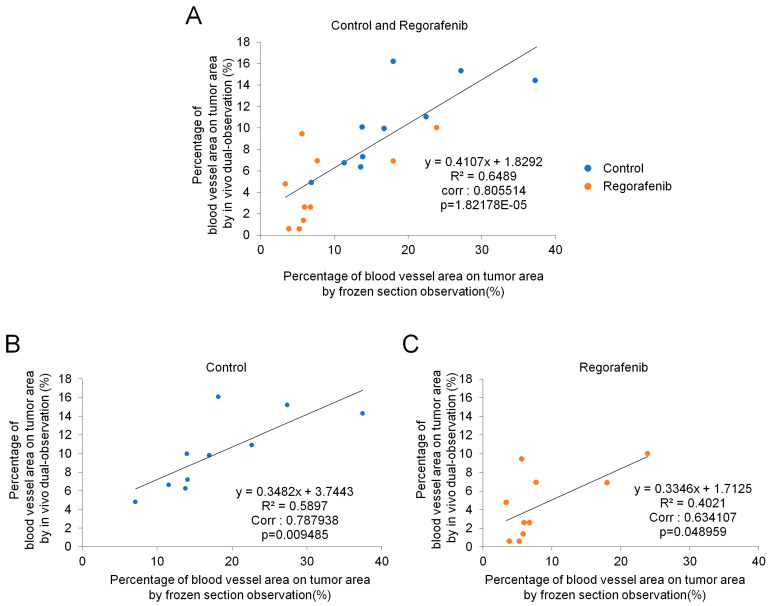
Correlation between in vivo dual-observation and frozen section observation of the percentage of blood vessel area on tumor area. Correlation with values in the (**A**) control group and regorafenib groups, (**B**) control group, and (**C**) regorafenib group.

To further investigate whether the intratumoral blood vessels were similarly reduced along with tumor-surface blood vessels, we performed fluorescent microscopic analysis with frozen sections obtained from isolated tumors on Day 35 after comparative experiments for the control and regorafenib groups. In the frozen section analysis, regions with RFP signals were labeled tumor areas and those stained with FITC-dextran were regarded as blood vessel areas [22]. Figure 5A shows images of representative frozen sections observed with fluorescence microscopy, and Figure 5B shows the quantitative analysis. The percentage of intratumoral blood vessel areas in tumor areas in the regorafenib group was significantly lower than the control group (*p* < 0.05) (Figure 5B); these results were consistent with the observation of tumor-surface blood vessel areas on Day 35 in the comparative experiment for the control and regorafenib groups using in vivo dual-observation (Figure 4B). There was a positive correlation between intratumoral blood vessel areas observed via frozen section analysis (Figure 5B) and tumor-surface blood vessel areas observed via the in vivo dual-observation method (Figure 4B) on Day 35 in this model (Corr = 0.805514, *p* < 0.001 for all samples in the control and regorafenib groups; Corr = 0.787938, *p* < 0.01 for control group; Corr = 0.634107, *p* < 0.05 for regorafenib group) (Figure 6).

## 3. Discussion

In this study, we developed a novel in vivo dual-observation method to observe the tumor mass and tumor-surface vessels by using a subcutaneous submillimeter tumor model with human colorectal cancer HT-29-RFP cells and intravenously administering FITC-dextran as a fluorescent angiographic contrast agent. In conventional methods, in vivo xenograft mouse models of subcutaneous tumors are constructed via an injection of cancer cell suspension; however, this makes it difficult to construct submillimeter tumors because cancer cells cannot localize in very small regions [7,17]. Therefore, in this study, we made a subcutaneous submillimeter tumor model by transplanting a single spheroid cultured on biocompatible and biodegradable rGHM into an SCID mouse. Our previous study demonstrated that rGHM is involved in the uniformity of the tumor shape and the timing of submillimeter xenograft formation [21]. Further, our study showed that submillimeter subcutaneous tumors can be observed in vivo in this model by detecting RFP signals using a stereoscopic fluorescence microscope. The present study evidenced that the administration of FITC-dextran to this model enabled the simultaneous detection of the tumor mass and tumor-surface blood vessels. We compared bright-field and green fluorescence images (Figure 1D) of the tumor-surface blood vessels of isolated tumor samples and found them to be similar. This indicated that tumor-surface blood vessels can be evaluated by analyzing green fluorescence images of FITC-dextran obtained using a fluorescence stereomicroscope. We observed no reduction in body weight after FITC-dextran administration, suggesting that the in vivo dual-observation method had negligible adverse effects. This method was useful for evaluating the inhibition of tumor growth and the downregulation of tumor-surface blood vessels after regorafenib treatment against submillimeter tumors. Alternatively, more specific fluorescent probes, such as fluorescent antibodies targeting blood vessel endothelial cells [23], may provide even more precise images of blood vessels and suppress the background noise. Additionally, in vivo dual-observation and image analysis revealed the downregulation of angiogenesis factors, such as vascular extremities, segments, and meshes. Therefore, this method may be a useful tool to develop anti-angiogenesis agents against submillimeter tumors. 

A positive correlation was found between intratumoral blood vessel areas observed via frozen section analysis and tumor-surface blood vessel areas observed via in vivo dual-observation. This suggests that the in vivo dual-observation of tumor-surface blood vessel areas might predict intratumoral vasculature; hence, this method would be helpful in elucidating angiogenesis in growing submillimeter tumors. For the correlation analysis, we observed significant positive correlations between intratumoral blood vessel areas and tumor-surface blood vessel areas in the control and regorafenib groups individually and together (Figure 6). The regorafenib group showed slightly lower Corr than the control group. This may be because some tumors showed smaller vessel areas in the tumor surfaces than in intratumoral areas owing to the effect of the regorafenib treatment. Interestingly, this method enabled the detection of tumor-surface vascular extremities, segments, and meshes, indicating the early stages of new blood vessel formation. This feature of this method makes it advantageous over other conventional methods for observing intratumoral vasculature in the tumor mass [24], thereby expanding our understanding of early tumor angiogenesis for the purpose of developing effective drugs against it. Currently, several approaches for the in vivo assessment of the tumor mass and vessels have been reported with specific devices, such as the newly developed photoacoustic 3D imaging system [25] and ultrahigh-resolution functional optical coherence tomography [26]. In contrast, this study uses a highly versatile stereoscopic fluorescence microscope, which provides a useful observation method. Notably, our methods can clearly distinguish the boundaries between the tumor mass and vessels with fluorescence, compared with other methods.

Colorectal cancer is the second leading cause of cancer death worldwide [27], and the existence of metastasis is a significant poor prognostic factor [28,29]. Therefore, the detection and treatment of submillimeter tumors at early metastasis are critical for improving prognosis [30]. Preclinical studies have reported that angiogenesis is a critical switch to stimulate tumor growth in early metastatic colorectal cancer; further, hypoxia emerging within the submillimeter tumor mass induces growth factors, such as VEGF, via the hypoxia-inducible factor (HIF), leading to angiogenesis [31]. Previous studies have demonstrated that HT-29 spheroids mimicking in vivo submillimeter tumors show central hypoxic areas along with the expression of HIF-1 and VEGF [32,33]. Hence, this method using HT-29-RFP would be helpful to develop anti-angiogenesis agents against metastatic colorectal cancer. In this method, we used the human colorectal cancer cell line HT-29, which has been well-studied and characterized in biology and genomics [32,34], to construct submillimeter xenografts. In addition, we used the HT-29 cell line, showing a strong RFP expression (HT-29-RFP) for the accurate detection of the submillimeter tumor mass, as established in a previous study [21]. Therefore, the HT-29-RFP cell line would be useful in the in vivo dual-observation of submillimeter tumors and in understanding the cellular drug response for drug development. 

In the present study, we used regorafenib as an anti-angiogenesis agent to evaluate the usefulness of this method. Regorafenib is a biaryl-urea compound, functioning as an oral multikinase inhibitor of angiogenic (VEGF R1-3, tyrosine kinase with immunoglobulin-like and epidermal growth factor-like domains, or TIE2), stromal (PDGF-β, fibroblast growth factor receptor 1), and oncogenic (RET, KIT, BRAF) receptor tyrosine kinases [35]. Regorafenib is FDA-approved for the treatment of metastatic colorectal cancer patients in refractory to standard chemotherapy. Anti-angiogenesis therapy is generally known to reduce nutritional and oxygen supply and inhibit tumor growth by reducing tumor blood vessels [36]. Previous preclinical studies have shown that regorafenib induces the reduction of blood vessels in colorectal cancer tumors [15,17]. This study observed that regorafenib treatment, which started at submillimeter-sized tumors, reduced blood vessels and inhibited tumor growth without toxicity. This suggests that regorafenib would also be effective in inhibiting the growth of submillimeter tumors at early metastasis and suppressing tumor progression in colorectal cancer. Such strategies employing anti-angiogenesis therapy against submillimeter tumors at early metastasis are also effective in other cancers. Future studies should focus on applying this method to other types of cancer cell lines to make a promising tool for anti-angiogenic development. Additionally, recent studies reported that patient-derived tumor xenografts using patient-derived cells or tissues collected from surgery are ideal candidates for mimicking in vivo tumors in patients [37]; therefore, the method developed in this study could also be used to construct patient-derived submillimeter tumor xenografts and observe their early angiogenesis. Moreover, previous clinical studies have observed in patients that new blood vessels are formed by branching off from the nearest blood vessels toward the growing tumor [9,10,38]. Despite having similar morphologies, the origins of species are different between tumor cells and blood vessels in this model; that is, the tumor cells were derived from humans, whereas the blood vessels were derived from mice [39]. Hence, it is necessary to consider the difference in biological and genomic features when using this model. 

## 4. Materials and Methods

### 4.1. Cell Cultivation and Culture of Spheroids on rGHM Arrays

Human colorectal carcinoma HT-29 cells were obtained from the American Type Culture Collection. HT-29 cells were stably transfected with RFP lentivirus (Lenti-Red, LG502; Biogenova, Rockville, MD, USA) reported previously [21]. In this study, a clone that strongly expressed the RFP, denoted as HT-29-RFP, was used for convenient in vivo detection [21]. Dulbecco’s Modified Eagle Medium (DMEM) (Wako Pure Chemicals Industry, Ltd., Osaka, Japan) supplemented with 10% fetal bovine serum was used as the culture medium to cultivate HT-29-RFP cells. Cells were cultured in a humidified atmosphere with 5% CO_2_ at 37 °C. Exponentially growing cells detached from the plates with trypsin were used. The number of viable cells was determined using the Trypan blue dye-exclusion method.

To construct spheroids of HT-29-RFP, the rGHM arrays were constructed using a radiation-molding method and pretreated as described previously [40]. Briefly, gelatin (porcine skin, Type A, G1890; Sigma–Aldrich, St. Louis, MO, USA) was dissolved in deionized water at 10% *w*/*w* at 50 °C and poured into 60 mm cell culture dishes. Micropatterned flexible polydimethylsiloxane molds loaded onto a polydimethylsiloxane film were placed on top of the gelatin solution. The samples were kept at 20 °C overnight, after which physical gelation was allowed. Further, irradiation of the samples was conducted using ^60^Co γ-ray (^60^Co No. 2 Irradiation Facility of Takasaki Institute for Advanced Quantum Science) in the air at 15–20 °C at 15 J/g (J/g = kGy) to construct sterilized rGHM arrays of 20 round-bottomed microwells (800 μm each in diameter and depth). The rGHM arrays were preincubated with 5 mL of phosphate-buffered saline at 50 °C for 2 h, exchanging the solution twice, and then incubated with 5 mL DMEM at 37 °C for 30 min, exchanging the medium twice.

To obtain spheroids, 2.0 × 10^6^ HT-29-RFP cells in 5 mL culture medium were seeded onto each rGHM array and cultured for 7 d. The spheroids were immediately used for transplantation to mice. They were observed using an inverted microscope (Olympus BX43; Olympus, Tokyo, Japan) equipped with a camera system (Olympus DP21; Olympus, Tokyo, Japan).

### 4.2. Ethical Approval

All animal experimental procedures were approved by the Animal Ethics Committee of the National Institutes for Quantum and Radiological Science and Technology (QST, Chiba, Japan) in 2018 (approval no. 13-1022-7) and conducted according to the institutional guidelines.

### 4.3. A Xenograft In Vivo Mouse Model of Subcutaneous Submillimeter Tumor

To construct a xenograft in vivo mouse model of a subcutaneous submillimeter tumor, HT-29-RFP spheroids cultured on rGHM array were used. Six-week-old female severe combined immunodeficiency (SCID) (CB17.Cg-Prkdc^scid^Lyst^bg-J^/CrlCrlj) mice obtained from the Jackson Laboratory Japan (Kanagawa, Japan) were used after acclimation for at least one week. Mice were fed a purified diet (AIN-93M; TestDiet, St. Louis, MO, USA) during the experiments to avoid the effect of diet on abdominal autofluorescence detected via in vivo fluorescence microscopic imaging and watered ad libitum. The spheroids were transplanted along with rGHM to mice as described previously [21]. Briefly, for the transplantation of spheroids, a 5 mm square of rGHM with a single spheroid located in the center was subcutaneously transplanted onto the right flank of the mouse through an incision. Mice were anesthetized with isoflurane, shaved, and disinfected at the surgical site before transplantation. The incision was closed with sutures. The subcutaneous submillimeter tumor model was obtained 21 d after transplantation.

### 4.4. Establishment of In Vivo Dual-Observation of Tumor Mass and Tumor-Surface Blood Vessels with Subcutaneous Submillimeter Tumor Model

To achieve in vivo dual-observation of tumor mass and tumor-surface blood vessels for submillimeter tumors, fluorescein-conjugated dextran (FD150S, Sigma-Aldrich, St. Louis, MI, USA) (FITC-dextran), as a fluorescent angiography agent, was administered into the obtained subcutaneous submillimeter tumor model. FITC-dextran of 150 KD (100 μg/100 μL) was intravenously injected in each mouse five minutes before observation. The previous study has shown that FITC-dextran of 150 KD, which was used in this study, shows very low vascular permeability in mice [41]. During in vivo observation, mice were anesthetized with isoflurane. Red fluorescent images for RFP signals from tumor mass and green fluorescent images for FITC signals from blood vessels of tumor mass along with bright-field images were obtained at regions of spheroid transplantation using a stereoscopic fluorescence microscope (MZ16F; Leica, Wetzlar, Germany) equipped with a camera system (DFC310FX; Leica, Wetzlar, Germany). This method was applied to the subcutaneous submillimeter tumor model on Day 21 after spheroid transplantation, as our previous study [21] showed that submillimeter tumors appear at this point. Images of in vivo dual-observation of tumor mass and tumor-surface blood vessels were obtained from submillimeter tumors on Day 21 and, thereafter, on Days 28 and 35 (*n* = 5). Mice were weighed, and their general health status was monitored twice a week. Mice were euthanized on Day 35, and tumors were isolated to observe the distribution of tumor-surface blood vessels by obtaining bright-field images using a stereoscopic fluorescence microscope and by obtaining the green fluorescence images of FITC-dextran for comparison. 

### 4.5. Comparative Experiment with Regorafenib

Further, to evaluate the usefulness of in vivo dual-observation for developing anti-angiogenesis agents against submillimeter tumors, a comparative experiment was performed involving mice treated with anti-angiogenesis agent regorafenib and a vehicle control. The mice of subcutaneous submillimeter tumor model described above were randomized into 2 groups. The first group (*n* = 10) was treated daily orally with regorafenib (Tokyo Kasei, R0142, Tokyo, Japan) (regorafenib group). Regorafenib was applied at a dose of 10 mg/kg body weight dissolved in polypropylene glycol, PEG400, Pluronic F68, and water (34:34:12:20), as described previously [42]. The second group (*n* = 10) received a daily oral administration of the vehicle polypropylene glycol, PEG400, Pluronic F68, and water (34:34:12:20) (control group). In vivo dual-observation of tumor mass and tumor-surface blood vessels was performed on Days 21, 28, and 35. Mice were weighed, and their general health status was monitored twice a week. Mice were euthanized on Day 35, and tumors were isolated, weighed, and subjected to fluorescent microscopic analysis with frozen sections.

### 4.6. Image Analysis on Tumor Volume and Angiogenesis Parameters

The images were digitalized, saved in an uncompressed tagged image file format (TIFF) for further analysis, and converted to a black-and-white binary image by setting the image threshold. The threshold-selected image parts were analyzed using ImageJ software version 1.53 (National Institutes of Health Image program) on a Windows operating system. The tumor size parameters (major length, minor length, and area) were measured using red fluorescent images. Tumor volume was calculated using the following equation: tumor volume = major length × minor length^2^ × π/6 [43,44]. The area of tumor-surface blood vessels was measured using green fluorescence images. Percentage of blood vessel area (%) was calculated using the following equation: Percentage of blood vessel area (%) = blood vessel area (mm^2^)/tumor area (mm^2^) × 100. Quantitative analyses of angiogenesis parameters, such as vascular extremities, segments, and meshes, were conducted using the Angiogenesis Analyzer plugin for ImageJ (National Institute of Health, Bethesda, MD, USA) (http://image.bio.methods.free.fr/ImageJ/?Angiogenesis-Analyzer-for-ImageJ, accessed on 27 September 2022) on tumor-surface blood vessels. 

### 4.7. Intratumoral Analysis via Fluorescence Microscopy with Frozen Sections

For further analysis of in vivo dual-observation of comparative experiment with regorafenib, fluorescent microscopic analysis with frozen sections was performed. The isolated tumors were embedded with an optimal cutting temperature compound (4583; Tissue-Tek, Zoeterwoude, The Netherlands) and frozen with dry ice to make frozen sections. The frozen tumors were halved, cutting surfaces were flattened, and 6 µm-thick frozen sections were obtained using a cryostat (Cryocut 1800; Leica, Wetzlar, Germany). These sections were observed with a fluorescence microscope (BZ-X810; Keyence, Osaka, Japan). RFP signals were detected from tumor and blood vessel areas stained with FITC [22] within tumors. The percentage of FITC-stained blood vessel area within the tumor area was calculated.

### 4.8. Statistical Analysis

Data were expressed as the mean and standard deviation. *p*-values were calculated using a 2-tailed *t*-test for comparisons between two groups. *p*-values < 0.05 were considered statistically significant.

## 5. Conclusions

We developed a novel method for the in vivo dual-observation of the tumor mass and tumor-surface blood vessels with a subcutaneous submillimeter tumor xenograft mouse model of human colorectal cancer HT-29-RFP cells with the intravenous administration of FITC-dextran as a fluorescent angiography agent. This method would be useful for developing anti-angiogenesis agents against submillimeter tumors at the early metastasis stage of colorectal cancer.

## Data Availability

The datasets used during the present study are available from the corresponding author upon reasonable request.
